# From reducing cognitive load to sustaining participation: psychological mechanisms, evidence, and stage-matched pedagogy for university tennis beginners

**DOI:** 10.3389/fpsyg.2026.1940031

**Published:** 2026-10-16

**Authors:** Jing Wang, Paramasivam Muthusamy

**Affiliations:** 1 Shanxi Institute of Science and Technology, Shanxi, China; 2 Faculty of Education and Liberal Arts, INTI International University, Nilai, Negeri Sembilan, Malaysia

**Keywords:** autonomous motivation, cognitive load, multidimensional engagement, nonlinear pedagogy, self-efficacy, stage-matched pedagogy, sustained participation, university tennis beginners

## Abstract

University tennis beginners face simultaneous technical, perceptual, tactical, and psychological demands that may exceed their attentional resources, challenge perceived competence, and diminish engagement. This mini-review synthesizes evidence on cognitive load, self-efficacy, autonomous motivation, multidimensional engagement, ecological dynamics, and beginner tennis pedagogy to explain how instructional design can support both skill acquisition and sustained participation. Evidence on equipment scaling and game-based learning indicates that low-compression balls, smaller courts, simplified rules, and cooperative tasks can increase the number of playable exchanges, enjoyment, and early confidence, although direct tests of cognitive load reduction are scarce and university-specific evidence remains limited. Constraints-led practice can then connect stroke production with perception, positioning, decision-making, and tactical intention, whereas selective technical instruction is most useful when a diagnosable movement feature restricts accuracy, consistency, or transfer. On this basis, this review proposes an evidence-informed, stage-matched (readiness-informed) conceptual framework that organizes individually supported instructional functions into a diagnostic, skill-specific, overlapping, and reversible structure rather than an empirically validated sequence: Stage 1 reduces entry barriers and builds credible mastery experiences; Stage 2 develops adaptive exploration and transfer; and Stage 3 refines limiting technical features while progressively restoring standard conditions. Engagement is positioned as a proposed mediating process linking psychological mechanisms to learning and participation. The framework extends evaluation beyond immediate performance toward retention, transfer, confidence under challenge, and sustained participation as an important but currently undertested outcome. Controlled and longitudinal studies are needed to evaluate the proposed mechanisms, pathways, and boundary conditions across diverse university settings.

## 1 Introduction

University physical education should develop motor competence while fostering enjoyment, personal meaning, and lifelong involvement in physical activity (Ennis, 2017). Tennis supports this aim because it can be played throughout one’s life; however, it remains demanding for non-athlete university beginners who often have limited experience and goals centered on health, enjoyment, and social participation (Ion-Muşat and Tupan, 2022). For this population, early instructional experiences can influence not only skill development but also confidence and willingness to remain engaged, although sustained participation is shaped by broader environmental opportunities. Continued involvement also depends on time and environmental opportunities, so initial interest alone may not ensure sustained participation (Deelen et al., 2018).

Beginners must coordinate strokes, footwork, ball-flight perception, timing, positioning, and tactical decisions. When these demands are introduced simultaneously through standard equipment, full courts, formal rules, and detailed explanations, working-memory capacity can be exceeded (Paas et al., 2003; Sweller, 2011). Repeated failure can weaken self-efficacy and persistence (Bandura, 1997), whereas environments that support autonomy, competence, and relatedness are more likely to foster self-determined participation (Deci and Ryan, 2000; Ntoumanis, 2001).

Technique-focused instruction can provide demonstrations, feedback, and targeted correction, although approaches relying predominantly on prolonged isolated drilling may reduce opportunities for representative play among adult beginners (Hublin et al., 2021). Quick and Easy Tennis instead uses low-compression balls, smaller courts, lighter rackets, simplified rules, and game-like tasks to create earlier success (Crespo et al., 2004; Vasil, 2005). Scaled equipment can improve rally success, control, enjoyment, commitment, and self-efficacy, although much of this evidence comes from children (Buszard et al., 2016; Piquer-Piquer et al., 2025). Non-linear pedagogy complements these modifications by manipulating task constraints to encourage exploration and adaptation (Renshaw et al., 2010; Chow, 2013; Hewitt et al., 2018), while staged exposure to standard conditions can support later refinement (Ke and Chano, 2024).

Nevertheless, tennis research has seldom integrated cognitive load, competence beliefs, motivation, and multidimensional engagement within a single explanatory framework, or examined whether classroom motivation extends beyond formal instruction (Fredricks et al., 2004; Lonsdale et al., 2009).

Rather than seeking a universally superior teaching method, this review examines how different pedagogical functions become more or less appropriate as learner readiness changes. Accordingly, technique instruction, modified conditions, and constraints-led practice are considered in terms of their instructional functions rather than as competing pedagogical categories. The distinctive contribution of this review is to synthesize cognitive load, self-efficacy, autonomous motivation, multidimensional engagement, and ecological motor learning within a stage-contingent framework. The model proposes that modified conditions primarily reduce entry barriers and may reduce unnecessary task demands while increasing opportunities for successful action–outcome experiences. These early mastery experiences are expected to strengthen perceived competence and self-efficacy, thereby supporting subsequent engagement and adaptive learning. Constraints-led practice increasingly supports adaptive perception and decision-making, and selective technical guidance becomes valuable when a diagnosable movement feature restricts performance. It therefore extends evaluation beyond immediate success to engagement, retention, transfer, and continued participation.

This mini-review examines not which tennis teaching method is universally superior, but how different instructional functions may contribute to learning and sustained participation-related outcomes through proposed psychological processes. We propose that equipment scaling and task simplification may first reduce perceived task difficulty and cognitive demand; where cognitive load is not directly measured, this link is treated as theoretically inferred rather than empirically established; mastery experiences and informational feedback shape perceived competence and task-specific self-efficacy; and autonomy-supportive structure influences need satisfaction and autonomous motivation. These proximal psychological experiences are expected to enhance behavioral, emotional, cognitive, and agentic engagement, which is positioned as a proposed mediator of skill acquisition, retention, transfer, and continuation intention. Prior experience, initial confidence, instructional conditions, and environmental opportunities are treated as boundary conditions. The resulting framework generates testable propositions while distinguishing direct psychological evidence from mechanism-consistent findings and theoretical inferences.

## 2 Scope and conceptual foundations of the review

### 2.1 Review scope and evidence selection

This mini-review focuses on non-athlete university tennis beginners. The scope was defined to identify and synthesize evidence relevant to instructional design, psychological mechanisms, and participation-related outcomes in this population. Studies conducted among university students who had little or no prior tennis experience were treated as direct evidence, whereas adult-beginner studies conducted outside university settings were considered closely related, but indirect, evidence. Findings from children, school physical education, other novice populations, and general motor-learning research were used cautiously to inform mechanisms, instructional principles, or boundary conditions rather than population-specific effects. Broader reviews were used to distinguish technique-centered, modified, and learner-centered approaches (Unierzyski and Crespo, 2007; Hewitt et al., 2018; Hublin et al., 2021; Ke and Chano, 2024).

A focused literature search was conducted in Scopus, Web of Science Core Collection, SPORTDiscus, and PsycINFO, covering publications from database inception to July 2026. Search strategies combined terms related to tennis or racket-sport instruction, novice learning, equipment modification, non-linear or game-based pedagogy, cognitive load, self-efficacy, motivation, engagement, retention, transfer, and participation using AND/OR combinations adapted for each database. The database-specific search strategies are provided in Supplementary Table S1.

The literature search and initial screening were performed by one author. Titles and abstracts were screened for relevance, followed by a full-text evaluation based on the predefined scope and eligibility criteria. A second author reviewed studies with uncertain relevance, ambiguous eligibility, or any discrepancies during the selection process, which were resolved through discussion and consensus.

Empirical studies and reviews that addressed novice learning and at least one instructional, psychological, skill-related, or participation-related outcome relevant to the proposed framework were included. Because this review aimed to develop a conceptual framework rather than estimate intervention effect sizes, studies were selected for their relevance to underlying mechanisms and instructional principles rather than statistical comparability. Studies that focused primarily on trained or elite athletes or unrelated outcomes were excluded. Key evidence sources informing the framework are summarized in Supplementary Table S1.

The search concepts were specified prospectively around novice tennis instruction and the psychological, learning, and participation constructs described above, whereas the three-stage framework was developed iteratively during evidence synthesis and was not used as an eligibility criterion.

Given the heterogeneity in populations, interventions, and outcomes, evidence was synthesized narratively rather than quantitatively. Evidence was qualitatively weighted according to study design, population relevance, intervention comparability, outcome measurement, and the inclusion of retention or transfer assessments. Greater interpretive emphasis was placed on controlled studies, validated measures, retention or transfer assessments, and university or adult-beginner samples. Evidence from children or less comparable contexts was treated as supportive rather than population-specific, and performance improvements were not interpreted as evidence of reduced cognitive load or enhanced competence unless these constructs were directly measured. A concise summary of key empirical studies informing the framework is provided in Supplementary Table S1, including population characteristics, instructional approaches, major outcomes, assessment of retention or transfer, direct measurement of psychological constructs, and principal limitations. The table also indicates key methodological limitations of the available evidence, including population differences, limited psychological measurement, and restricted assessment of retention or transfer.

### 2.2 Integrative conceptual framework

Four complementary perspectives guide this review. These perspectives are used as complementary explanatory lenses rather than as a fully validated causal model. Cognitive load theory explains why standard equipment, full courts, complex rules, and multiple technical cues can exceed beginners’ working-memory capacity. Simplification reduces unnecessary extraneous processing, although task difficulty must rise as learners become more proficient (Mayer and Moreno, 2003; Paas et al., 2003; Guadagnoli and Lee, 2004; Sweller, 2011). Self-efficacy and self-determination theories explain whether students feel capable and are willing to invest effort. These perspectives are therefore considered complementary: reducing unnecessary task demands creates conditions for successful performance experiences, while perceived competence and self-efficacy influence whether learners remain engaged when task demands progressively increase. Together, these mechanisms represent a proposed pathway in which instructional design shapes psychological experiences that subsequently support learning and participation-related outcomes. Mastery experiences can strengthen confidence, persistence, and self-regulation, while autonomy support, informative feedback, and social connection foster more self-determined motivation (Bandura, 1997; Deci and Ryan, 2000; Zimmerman, 2000; Ntoumanis, 2001; Schunk and DiBenedetto, 2020; Guay, 2022).

Engagement is proposed as a mediating process through which these psychological experiences influence learning. It encompasses behavioral participation, emotional involvement, cognitive investment, and agentic active contribution to instruction; disengagement may appear as withdrawal, frustration, or reduced attention (Fredricks et al., 2004; Standage et al., 2005; Skinner et al., 2009). Ecological dynamics adds a motor-learning perspective by explaining how interactions among the learner, task, and environment support functional movement, decision-making, adaptation, and transfer (Newell, 1986; Araújo et al., 2006; Renshaw et al., 2010; Pinder et al., 2011; Chow, 2013; Button et al., 2020).

Figure 1 integrates these perspectives into a proposed conceptual pathway from instructional design through proximal psychological mechanisms and multidimensional engagement to skill and participation outcomes. The positioning of engagement as a mediator reflects a theoretical synthesis rather than an empirically confirmed mediation model. The model also recognizes reciprocal reinforcement because successful performance can strengthen competence and self-efficacy, while learner characteristics, teaching conditions, and environmental opportunities can modify each stage of the pathway. These reciprocal relationships are conceptual considerations that influence implementation and learner responses rather than empirically established feedback loops. Tasks, instructional support, and assessments should therefore remain aligned with learner readiness and intended outcomes rather than being organized around a single fixed method (Biggs, 1996).

**Figure 1:**
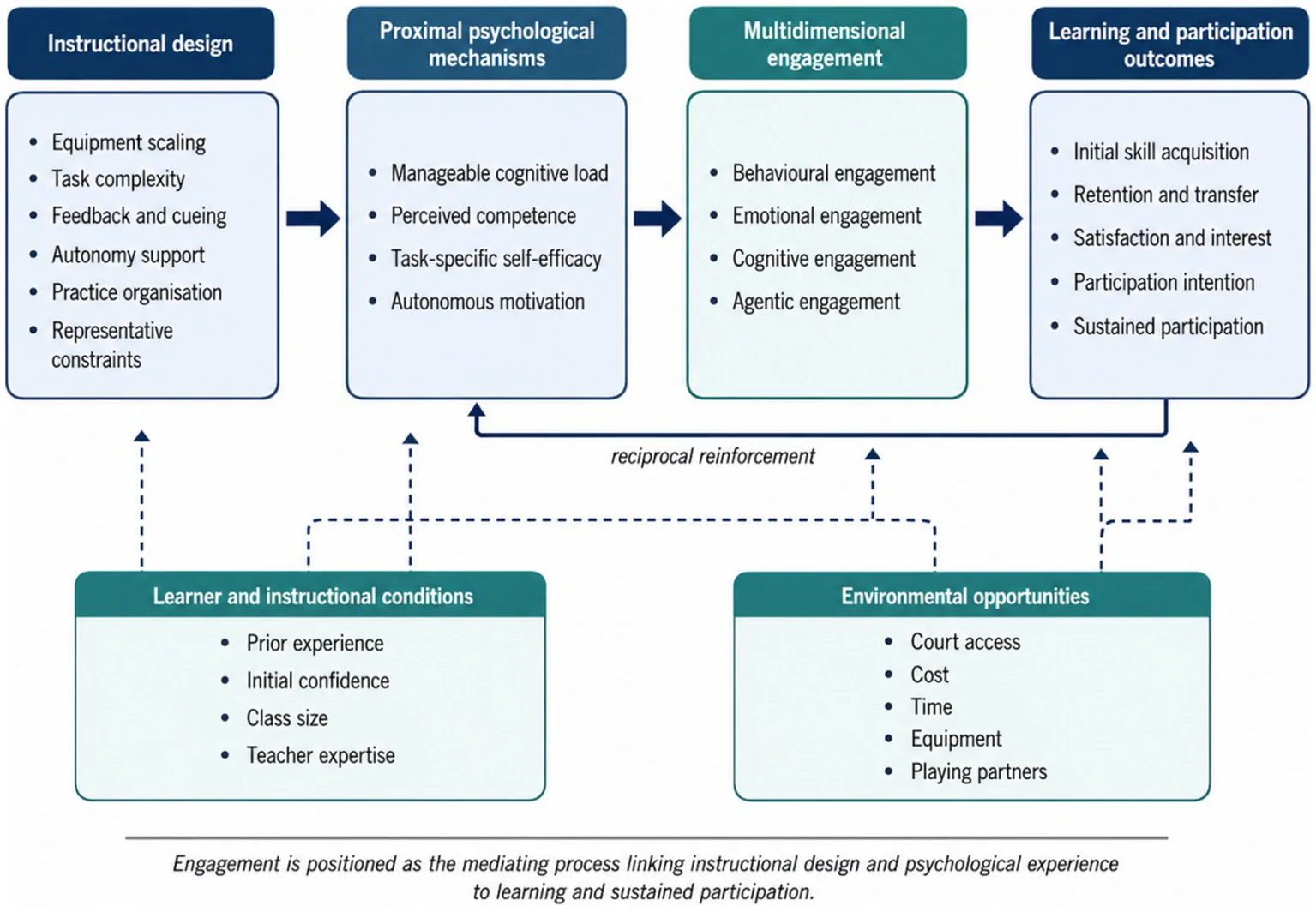
An integrated pathway from instructional design to skill development and sustained tennis participation. Cognitive load reduction and engagement are presented as proposed mechanisms within the framework; cognitive load reduction is inferred from task simplification and modified conditions, whereas engagement as a mediating process requires further empirical testing in university tennis beginners.

## 3 Psychological mechanisms

The mechanisms discussed below are proposed explanatory pathways that describe how instructional conditions influence beginner learning experiences; they should not be interpreted as independently established causal effects within university tennis populations.

### 3.1 Manageable challenge and cognitive load

University tennis beginners must coordinate technical, perceptual, spatial, and tactical information before stable movement representations have formed. Standard balls, full courts, complex rules, and multiple corrective cues can therefore exceed working memory capacity, leaving little attention for ball flight, timing, and action consequences (Mayer and Moreno, 2003; Paas et al., 2003; Sweller, 2011). Quick and Easy Tennis improves task–learner fit by slowing the ball, reducing space and force demands, and increasing the number of playable exchanges. These changes generally support early performance, rally success, enjoyment, engagement, and self-efficacy. Through increased opportunities for successful action–outcome experiences, these modifications may contribute to perceived competence and may reduce unnecessary attentional demands. However, performance, enjoyment, or self-efficacy improvements do not themselves demonstrate reduced cognitive load; where cognitive load or mental effort has not been measured directly, reduced cognitive demand should be regarded as a theoretically plausible mechanism rather than an established effect (Farrow and Reid, 2010; Buszard et al., 2016; Piquer-Piquer et al., 2025).

Simplification should not remove challenge. Technique-based instruction can reduce uncertainty through clear demonstrations, appropriately selected cues, and outcome-related feedback. However, when instruction becomes dominated by excessive correction or isolated internal-focused control, it can increase conscious monitoring and divert attention from intended performance outcomes. External-focus attentional instructions are often more effective, although effects vary by task and learner (Wulf and Lewthwaite, 2016; Chua et al., 2021). Constraints-led practice offers another route: modifying court size, targets, scoring, or partner distance can narrow the search space while preserving representative perception–action relations (Renshaw et al., 2010; Pinder et al., 2011; Chow, 2013). The goal is an optimal challenge that remains informative without becoming overwhelming or trivial (Guadagnoli and Lee, 2004).

### 3.2 Competence, self-efficacy, and autonomous motivation

Early experiences shape whether students regard tennis as learnable. Successful contact, directional control, and short rallies provide mastery experiences that can strengthen perceived competence and task-specific self-efficacy (Bandura, 1997). Informational feedback should connect success to controllable actions rather than vague praise or social comparison; arrangements that protect competence and offer learner control may also improve learning (Chiviacowsky et al., 2012; Wulf and Lewthwaite, 2016). Stronger efficacy supports persistence, strategic effort, and recovery after errors, while improved performance supplies further mastery evidence, creating a reciprocal confidence–learning cycle (Zimmerman, 2000; Schunk and DiBenedetto, 2020). Within the proposed framework, this cycle helps explain how early instructional success contributes to continued engagement, although the strength and direction of these relationships require further empirical testing.

Motivation also depends on how instruction is communicated. Meaningful rationales, bounded choice, acknowledgment of difficulty, supportive feedback, and positive peer interaction can satisfy the basic psychological needs for autonomy, competence, and relatedness without removing teacher structure (Deci and Ryan, 2000; Reeve and Cheon, 2021; Guay, 2022). Physical education evidence links these conditions to autonomous motivation, engagement, performance, wellbeing, and intentions to remain active (Ntoumanis, 2001; Standage et al., 2005; Behzadnia et al., 2018; Vasconcellos et al., 2020). Non-linear pedagogy may further support guided agency by allowing learners to explore functional solutions within carefully selected constraints (Chow, 2013; Button et al., 2020).

### 3.3 Risks of poor calibration

Modified or learner-centered instruction is not automatically supportive. Prolonged simplification can induce boredom and weak transfer; overly competitive games may threaten competence; excessive correction can fragment attention; and unstructured exploration can leave beginners confused. Controlling language, public criticism, and normative comparison can frustrate psychological needs even when tasks are technically appropriate (Bartholomew et al., 2011; Haerens et al., 2015).

Instruction should therefore be continuously calibrated. Instructors must progress task demands, preserve meaningful choice within a clear structure, use selective feedback, and monitor indicators of disengagement, including withdrawal, frustration, anxiety, confusion, and declining attention. Psychological benefits should remain plausible mechanisms rather than assumed consequences of any method, because direct tests of the pathway from instructional design to cognitive load, efficacy, motivation, and engagement remain scarce. Accordingly, these mechanisms are best interpreted as hypotheses guiding instructional design and future research rather than established explanatory pathways.

## 4 Engagement and outcomes

### 4.1 Engagement as a proposed mediating process

In this review, engagement is considered a proposed explanatory process linking psychological experiences to learning behaviors and participation-related outcomes, rather than an established causal mechanism.

Manageable task demands, competence beliefs, and autonomous motivation may influence learning partly through sustained involvement. Within the proposed framework, engagement is conceptualized as a potential mediating process linking proximal psychological experiences with learning and participation outcomes, although direct mediation evidence in university tennis remains limited. Engagement is therefore proposed as a mediating process rather than treated only as a secondary indicator of enjoyment. In this framework, engagement represents the learner’s ongoing behavioral, emotional, cognitive, and agentic involvement that may help translate favorable instructional and psychological conditions into learning-related responses. Behavioral engagement concerns active practice and persistence; emotional engagement includes interest, anxiety, and frustration; cognitive engagement involves attention, decision-making, and strategic adjustment (Fredricks et al., 2004; Reeve, 2012). Disaffection may emerge as withdrawal, boredom, helplessness, or inattention before participation visibly declines (Skinner et al., 2009). Students tend to invest more effort when tasks are purposeful, achievable, and supported by clear structure and meaningful choice (Jang et al., 2010). Physical education evidence linking self-determined motivation with activity during compulsory and free-choice periods is consistent with this proposed pathway, although direct mediation tests remain scarce in university tennis (Standage et al., 2005; Lonsdale et al., 2009).

### 4.2 Skill acquisition and transfer

For beginners, functional skill acquisition involves reliable contact, directional control, recovery, and elementary rallying. Scaled equipment and cooperative tasks increase playable balls and complete perception–action–feedback cycles, generally improving early performance and practice density (Farrow and Reid, 2010; Buszard et al., 2016). Game-based instruction can make tennis meaningful earlier than prolonged isolated drilling (Hublin et al., 2021), although heterogeneous measures and limited retention tests restrict claims about lasting advantage (Piquer-Piquer et al., 2025). Accordingly, immediate performance gains should be distinguished from demonstrated retention, transfer, and long-term participation effects.

Later refinement requires selective technical feedback linked to observable ball outcomes, without excessive body-focused control (Wulf and Lewthwaite, 2016; Hewitt et al., 2018). Transfer depends on representative tasks preserving information about trajectory, space, timing, and other players (Pinder et al., 2011; Hewitt et al., 2018). Purposeful variability can promote adaptable solutions when matched to current capability (Renshaw et al., 2010; Ranganathan and Newell, 2013; Button et al., 2020). Progression toward standard equipment and scoring should therefore alter constraints gradually to maintain optimal challenge (Guadagnoli and Lee, 2004).

### 4.3 Sustained participation

University tennis courses should develop skills and dispositions usable beyond formal instruction (Beni et al., 2017; Ennis, 2017). Course engagement, continuation intention, and actual participation must nevertheless be distinguished from one another. Enjoyment and confidence may strengthen intentions, but time, cost, facilities, and partner availability can prevent behavior (Deelen et al., 2018). Autonomous motivation may transfer from physical education to leisure contexts when teaching supports competence, choice, and personal value (Hagger et al., 2003; Behzadnia et al., 2018). However, whether these instructional and psychological processes translate into sustained participation in tennis remains insufficiently tested. Sustained participation should therefore be assessed through follow-up behavior, not intention alone, and understood as the joint product of psychological readiness, adaptable skill, and environmental opportunity. Within the present framework, sustained participation represents a proposed distal outcome that requires longitudinal investigation rather than an established consequence of any specific pedagogical approach.

## 5 Stage-matched (readiness-informed) framework

The three-stage framework is an evidence-informed synthesis rather than an empirically validated progression model. The term “stage” describes dominant instructional functions associated with changing learner readiness rather than a fixed developmental sequence. Learners may overlap across stages, and different skills within the same learner may require different instructional functions simultaneously. Available evidence supports individual instructional functions, including modified conditions, constraints-led practice, and selective technical guidance; however, evidence directly validating their proposed ordering or transition logic remains limited. It integrates findings on modified conditions, constraints-led practice, and selective technical guidance to match instructional functions to changing learner readiness (Farrow and Reid, 2010; Buszard et al., 2016; Hublin et al., 2021; Ke and Chano, 2024). Modified conditions are used primarily to facilitate early success, constraints-led tasks to support adaptation, and selective technical guidance to address limiting movement features. The stages may overlap, vary by skill, and reverse under novelty or pressure. Because the complete sequence and its transition thresholds have not been directly validated, the readiness and progression indicators in Table 1 should be interpreted as provisional instructional heuristics for adaptation and monitoring rather than fixed criteria for learner classification or progression.

**Table 1:** Proposed stage-matched (readiness-informed) pedagogy and provisional decision-support indicators for non-athlete university tennis beginners.

Stage and learner readiness	Mechanism-targeted instructional design	Proposed psychological pathway and engagement response	Teacher role, monitoring, and progression	Risks, boundary conditions, and corrective adjustment
Stage 1: Regulating load and protecting competence Readiness: Learners show inconsistent racket–ball contact, limited directional control, high mental effort, or anxiety about visible errors, and cannot yet sustain a short cooperative rally.	Function: Make tennis immediately playable while preserving meaningful challenge. Use low-compression balls, reduced courts, lighter rackets, simplified rules, large targets, cooperative feeding, short rally tasks, and small groups to increase successful contacts and reduce waiting time (Crespo et al., 2004; Vasil, 2005; Farrow and Reid, 2010; Buszard et al., 2016).	Proposed pathway: Lower perceived task difficulty and attentional competition → clearer action–outcome information and credible mastery experiences → perceived competence and task-specific self-efficacy → willingness to engage and persist. Expected response: Emotional safety, enjoyment, behavioral persistence, and willingness to re-engage after error. Performance and enjoyment benefits are more directly supported than cognitive-load mediation.	Use one principal outcome-focused cue, brief demonstrations, informational feedback, bounded choices, and progress-based evaluation. Monitor perceived mental effort, frustration, anxiety, re-engagement, contact reliability, directional control, and short-rally maintenance. Provisional indicators for increasing difficulty include stable contact, directional control, and short-rally maintenance without a marked increase in perceived effort, frustration, anxiety, or withdrawal (Bandura, 1997; Deci and Ryan, 2000).	Risks and boundaries: Excessive simplification, boredom, dependence on protected conditions, confidence restricted to simplified tasks, or competition that threatens competence. Adjustment: Change one constraint at a time, such as distance, target precision, ball speed, or movement demand; restore support if anxiety, frustration, or withdrawal rises.
Stage 2: Supporting autonomous exploration and adaptive confidence Readiness: Learners establish basic contact and maintain exchanges under relatively predictable conditions, leaving sufficient attention for positioning, ball information, and tactical problems.	Function: Connect stroke production with perception, decision-making, positioning, and tactical intention. Manipulate court dimensions, target zones, ball trajectories, partner positions, scoring rules, and tactical objectives. Use representative tasks, guided variability, and cooperative or semi-competitive problems that preserve perception–action information (Renshaw et al., 2010; Pinder et al., 2011; Chow, 2013).	Proposed pathway: Structured choice and guided exploration → autonomy support, strategic confidence, and attention to relevant information → cognitive and agentic engagement. Expected response: Willingness to test alternative solutions, explain simple tactical intentions, and remain engaged as uncertainty increases, supporting adaptable rather than condition-specific skill.	Design constraints that invite functional responses; use focused questions, occasional demonstrations, and selective cues when exploration stalls. Structure peer observation around ball outcomes, space, and tactical choices rather than technical judgment. Provisional indicators for increasing challenge include adaptation to changes in height, speed, direction, or court space, maintenance of rallies under reduced predictability, and the ability to explain simple tactical intentions (Araújo et al., 2006; Button et al., 2020).	Risks and boundaries: Random variability, excessive choice, unclear task purpose, unstructured trial and error, or competence threat under premature competition. Adjustment: Narrow the search space, clarify the tactical problem, reduce one source of uncertainty, or temporarily restore teacher guidance.
Stage 3: Developing self-regulation and confidence under challenge Readiness: Learners participate functionally in representative play, but an identifiable movement feature limits accuracy, consistency, efficiency, or adaptation to more standard conditions.	Function: Refine a specific technical constraint without displacing meaningful play. Use short blocks of purposeful repetition, external-focus cues, targeted demonstration, optional video feedback, and gradual transitions to faster balls, larger courts, and formal scoring; return adjustments quickly to representative tasks (Hodges and Franks, 2002; Ke and Chano, 2024).	Proposed pathway: Targeted, outcome-linked feedback with progressively reduced support → stable competence, deliberate cognitive engagement, self-monitoring, and independent correction. Expected response: Persistence and confidence under more standard or competitive conditions, with stable engagement as challenge increases.	Diagnose whether the feature genuinely restricts the intended outcome; correct one feature at a time and connect it to ball performance. Monitor retention after a period without practice, transfer across balls, courts, partners, and tactical demands, self-correction, and engagement under standard conditions. Provisional indicators for reducing instructional support include improvements that persist when continuous feedback is withdrawn and across increasingly representative conditions (Wulf and Lewthwaite, 2016; Chua et al., 2021).	Risks and boundaries: Excessive correction, internal attentional focus, decontextualised drilling, abrupt standardisation, feedback dependence, or competence loss. Adjustment: Reconnect technique to an external outcome, reduce feedback, restore representative play, reduce one constraint, or return temporarily to an earlier support condition.

Stages denote readiness-informed instructional functions rather than fixed developmental categories and may overlap, differ by skill, or reverse. Cited instructional principles are evidence-informed; psychological pathways are theoretically proposed; readiness and adjustment indicators are author-proposed decision-support heuristics rather than validated thresholds, diagnostic criteria, or a validated sequential protocol.

Planning therefore begins with instructional diagnosis: does the learner need fewer barriers, broader adaptability, or correction of a limiting movement feature? Equipment, constraints, feedback, and practice organization follow that function. The same student may refine the forehand, simplify the serve, and explore rally tactics simultaneously; thus, stage placement is both learner- and skill-specific.

The following subsections summarize each stage’s rationale; Table 1 supplies practical applications, transition indicators, and corrective responses.

### 5.1 Stage 1: reducing barriers and building confidence

At entry, standard tennis can exceed beginners’ perceptual, motor, and attentional resources. Low-compression balls, reduced courts, lighter rackets, simplified rules, and cooperative tasks improve task–learner fit and reduce task-related processing demands, although such cognitive-load reductions remain theoretically inferred unless measured directly (Paas et al., 2003; Crespo et al., 2004; Vasil, 2005; Sweller, 2011). By increasing playable contacts and short rallies, these modifications create mastery experiences that strengthen early self-efficacy (Bandura, 1997; Farrow and Reid, 2010; Buszard et al., 2016; Piquer-Piquer et al., 2025). Accordingly, instruction should be brief, equitable, and outcome-focused. Providing a single usable cue and bounded choice preserves structure while supporting competence, autonomy, and relatedness (Deci and Ryan, 2000; Ntoumanis, 2001). Potential indicators for progression include stable contact, repeatable short exchanges, and tolerance of modest variation without marked frustration.

### 5.2 Stage 2: adaptive exploration and skill transfer

Once basic control emerges, practice should connect stroke production with ball information, positioning, decision-making, and tactical purpose. Manipulating court size, targets, scoring, ball characteristics, and partner distance invites functional solutions while preserving representative perception-action information (Renshaw et al., 2010; Pinder et al., 2011; Chow, 2013). Learners should adapt to changing trajectories, spaces, and opponents rather than merely reproducing an isolated stroke (Araújo et al., 2006).

Exploration still requires pedagogical structure. Carefully chosen constraints, focused questions, and occasional demonstrations prevent variability from becoming random trial and error (Ranganathan and Newell, 2013; Button et al., 2020). Potential readiness indicators include adaptable stroke mechanics, simple tactical explanations, and sustained cognitive involvement as task predictability declines.

### 5.3 Stage 3: technical refinement and stabilization

Technical instruction is most useful when a recurring movement feature limits accuracy, consistency, or adaptation. Feedback should isolate one correctable feature, link it to an observable ball outcome, and avoid unnecessary internal-focused control (Wulf and Lewthwaite, 2016; Chua et al., 2021). Demonstration and explanation should address a defined performance problem, not impose an idealized technical form (Hodges and Franks, 2002).

Brief purposeful repetition may stabilize an adjustment, but the movement should quickly return to rallies or representative tasks. Transitions to standard balls, court dimensions, and scoring should generally be gradual, since abrupt standardization can compromise transfer and confidence (Ke and Chano, 2024). Technical refinement should extend, not displace, meaningful play (Hublin et al., 2021).

### 5.4 Transition heuristics and boundary conditions

The transition indicators in Table 1 are intended as provisional decision-support heuristics rather than empirically validated cut-offs or diagnostic criteria. They should therefore be interpreted as decision-support information for adjusting instructional functions rather than as criteria for progressing learners through a universal sequence. Decisions to increase, maintain, or restore instructional support should therefore draw on converging information from performance, adaptation, retention, or transfer, and engagement. Reliable contact, rally maintenance, retention, and transfer indicate readiness more convincingly than immediate practice success (Soderstrom and Bjork, 2015), whereas anxiety, confusion, boredom, or withdrawal signal poorly calibrated difficulty (Fredricks et al., 2004; Skinner et al., 2009). Guidance can recede with increasing proficiency and return when novelty rises (Kalyuga, 2007) while task difficulty should remain responsive to the learner’s current capabilities (Guadagnoli and Lee, 2004).

Implementation must accommodate learner differences, class size, equipment, instructor expertise, and interpersonal style. Clear rationales, meaningful choices, and actionable feedback preserve autonomy within structure (Reeve and Cheon, 2021). Fidelity concerns whether the intended function is achieved, not rigid procedural uniformity (Carroll et al., 2007). Because continuation also depends on court access, time, cost, and partners availability (Deelen et al., 2018), the framework should guide ongoing adjustment rather than operate as a fixed protocol.

## 6 Discussion

### 6.1 Synthesis and contributions

Evidence directly aligned with the target population remains limited but includes university beginner studies of game-based instruction and modified or staged learning conditions (Hublin et al., 2021; Kilit et al., 2023; Ke and Chano, 2024). Modified equipment and cooperative tasks can improve task–learner fit, playable exchanges, and early performance, and may reduce cognitive demands by simplifying task requirements; however, this cognitive mechanism is more often theoretically inferred than directly measured (Paas et al., 2003; Guadagnoli and Lee, 2004; Farrow and Reid, 2010; Sweller, 2011; Buszard et al., 2016; Piquer-Piquer et al., 2025). The proposed links to cognitive load, autonomous motivation, and multidimensional engagement therefore draw more strongly on indirect empirical findings and established psychological theory where university-specific evidence is unavailable (Bandura, 1997; Deci and Ryan, 2000; Fredricks et al., 2004; Reeve and Cheon, 2021).

The distinctive theoretical contribution of this framework is to match these psychological and motor-learning functions to changing learner readiness. Sustained participation should therefore be interpreted as a proposed distal outcome of the framework rather than an established effect of the reviewed pedagogical approaches. Quick and Easy Tennis, non-linear pedagogy, and technical guidance are therefore treated as complementary, stage-dependent functions rather than competing methods (Hublin et al., 2021; Ke and Chano, 2024). Importantly, the available literature supports the relevance of these instructional functions individually, whereas the proposed sequence of their introduction represents an integrative hypothesis that requires direct empirical testing. Progression is diagnostic, skill-specific, and reversible, drawing on control, adaptation, engagement, and tolerance of difficulty rather than immediate performance alone.

### 6.2 Practical implications

The following recommendations should be interpreted as evidence-informed teaching principles rather than a validated instructional protocol. In applying these evidence-informed principles, instructors should maximize meaningful hitting opportunities, limit the number of simultaneous technical cues, and adjust equipment, rules, and the level of uncertainty according to learner readiness. Feedback should connect one correctable feature to an observable ball outcome, while representative tasks preserve perception–action information and guided exploration (Pinder et al., 2011; Chow, 2013; Wulf and Lewthwaite, 2016; Hewitt et al., 2018; Button et al., 2020; Chua et al., 2021). Clear structure can coexist with meaningful choice, rationale, and learner control (Reeve and Cheon, 2021; Guay, 2022). Assessment should combine skill, transfer, cognitive load, self-efficacy, motivation, and multidimensional engagement rather than relying solely on immediate technical scores.

### 6.3 Limitations and future research

Direct evidence from university beginners remains limited, with small sample sizes, heterogeneous measures, few longitudinal designs, and frequent inference of psychological mechanisms from motor performance. As this review was designed as a focused narrative synthesis rather than a systematic review, evidence selection and qualitative weighting were not accompanied by formal risk-of-bias assessment or quantitative quality scoring. Evidence strength was instead assessed based on methodological characteristics and relevance to the target population; accordingly, the proposed framework should be regarded as an evidence-informed synthesis requiring independent empirical testing. Future studies should compare conventional, fully modified, and stage-matched instruction while examining the pathway from instructional design through psychological mechanisms and engagement to retention, transfer, satisfaction, and actual participation. Retention should be separated from practice performance (Soderstrom and Bjork, 2015), and behavior from intention, because access, time, cost, and partners may interrupt continuation (Sheeran and Webb, 2016; Deelen et al., 2018). Longer follow-up should examine reciprocal links between competence and participation and the development of enduring interest (Hidi and Renninger, 2006; Stodden et al., 2008).

## 7 Conclusion

University tennis instruction should develop usable skills without undermining confidence, motivation, or engagement. Stage-matched pedagogy coherently combines early simplification, adaptive exploration, and technical refinement while supporting lifelong participation goals (Beni et al., 2017; Ennis, 2017). However, the model remains evidence-informed rather than fully validated and requires controlled, longitudinal testing across diverse university settings.
