# Supplementary material for: From reducing cognitive load to sustaining participation: psychological mechanisms, evidence, and stage-matched pedagogy for university tennis beginners
Source: Front Psychol. 2026 Oct 16;17:1940031. doi: 10.3389/fpsyg.2026.1940031 (PMC13720767; doi:10.3389/fpsyg.2026.1940031)
Supplement: Supplementary file 1 [file Table_1.DOCX]

Table S1. Search strategies and summary of key evidence informing the review framework

Panel A. Database search strategies

| **Database** | **Search strategy (simplified)** | **Search period** |
| --- | --- | --- |
| Scopus | TITLE-ABS-KEY ((tennis OR "racket sport*") AND (beginner* OR novice OR "university student*" OR "adult beginner*") AND (instruction* OR pedagogy OR teaching OR "game-based learning" OR nonlinear OR "constraints-led" OR "equipment scaling" OR "modified equipment") AND ("cognitive load" OR self-efficacy OR motivation OR engagement OR retention OR transfer OR participation)) | Database inception to July 2026 |
| Web of Science Core Collection | TS=((tennis OR "racket sport*") AND (beginner* OR novice OR "university student*" OR "adult beginner*") AND (instruction* OR pedagogy OR teaching OR "game-based learning" OR nonlinear OR "constraints-led" OR "equipment modification") AND ("cognitive load" OR self-efficacy OR motivation OR engagement OR retention OR transfer OR participation)) | Database inception to July 2026 |
| SPORTDiscus | (tennis OR "racket sport*") AND (beginner* OR novice OR "adult learner*" OR university) AND (instruction* OR teaching OR pedagogy OR "game-based learning" OR "constraints-led approach" OR "modified equipment") AND (learning OR skill acquisition OR motivation OR self-efficacy OR engagement OR participation) | Database inception to July 2026 |
| PsycINFO | (tennis OR "racket sport*") AND (beginner* OR novice OR student* OR adult learner*) AND (instruction OR learning OR pedagogy OR motor learning) AND ("self-efficacy" OR motivation OR engagement OR "cognitive load" OR persistence OR participation) | Database inception to July 2026 |

| Panel B. Summary of key evidence sources informing the review framework | | | | | | |
| --- | --- | --- | --- | --- | --- | --- |
| **Study** | **Population** | **Instruction/intervention or focus** | **Main outcomes relevant to framework** | **Retention/transfer assessed** | **Psychological constructs directly measured** | **Key methodological limitations** |
| Farrow & Reid (2010) | Beginning tennis players | Equipment scaling (modified equipment conditions) | Improved early tennis skill acquisition and practice conditions | Limited | No direct measurement of cognitive load or self-efficacy | Skill-focused outcomes; limited psychological assessment |
| Buszard et al. (2016) | Children in sport contexts (systematic review) | Scaling equipment and play area | Modified conditions generally supported motor skill acquisition | Variable | Not directly assessed | Population differs from university beginners |
| Hublin et al. (2021) | Adult tennis beginners | Game-based teaching vs conventional training | Game-based approach improved tennis learning efficiency | Limited | Limited psychological measures | Adult sample rather than university-specific |
| Kilit et al. (2023) | Adult beginner tennis players | Ball compression and court-size modifications | Modified balls and courts influenced tennis skill learning | Limited | Not directly assessed | Short-term intervention; adult population |
| Ke & Chano (2024) | University students/general college context | Short tennis + standard tennis staged teaching approach | Feasibility of progressive transition toward standard tennis | Limited | Not directly assessed | Feasibility study; framework not fully validated |
| Piquer-Piquer et al. (2025) | Novice tennis populations (scoping review) | Equipment modification literature | Summarised effects of modified equipment on novice learning | Variable | Variable | Evidence heterogeneity across populations and designs |
| Crespo et al. (2004) | Tennis learners/coaching context | Game-based tennis teaching approach | Supported playable tasks and learner-centred instruction principles | Not directly examined | Not directly assessed | Practice-oriented rather than controlled research |
| Standage et al. (2005) | Physical education students | Self-determination theory-based PE context | Autonomous motivation associated with engagement-related outcomes | Not tennis-specific | Motivation directly measured | Indirect evidence from PE settings |
| Lonsdale et al. (2009) | Physical education students | Self-determined motivation in PE lessons | Linked motivation with physical activity participation | Not tennis-specific | Motivation directly measured | Context differs from tennis learning |
| Vasconcellos et al. (2020) | Physical education populations (meta-analysis) | Self-determination theory in PE | Supported relationships among autonomy support, motivation, and engagement | Not tennis-specific | Motivation-related constructs measured | Broad PE evidence, not tennis-specific |

**Note:** Studies were selected to represent the main empirical evidence supporting the conceptual framework. Evidence from university tennis beginners was prioritised where available; adult beginner, youth, physical education, and broader motor-learning studies were considered indirect evidence informing mechanisms or instructional principles. Psychological pathways and stage progression remain proposed mechanisms unless directly measured.
